# Evaluation of the Use of Highly Concentrated Autologous Platelet-Rich Plasma and Platelet-Rich Fibrin Membrane to Improve the Outcome in the Management of Severe Dry Eye Disease, Corneal Neurotrophic Ulcer and Corneal Burn

**DOI:** 10.7759/cureus.51794

**Published:** 2024-01-07

**Authors:** Aaliouet Hassan, Alain Telandro, Abouddihaj Barguigua, Mustafa Baba, Norbert Körber

**Affiliations:** 1 Ophthalmology, Casablanca Eye Center, Casablanca, MAR; 2 Ophthalmology, Ophtalmologue Le Cannet-des-Maures, Cannes, FRA; 3 Microbiology, Polydisciplinary Faculty, Sultan Moulay Slimane University, Beni Mellal, MAR; 4 Environment and Climate, Ecole Hassania des Travaux Publics, Casablanca, MAR; 5 Ophthalmology, Augenzentrum Josefstr, Köln, DEU

**Keywords:** dry eye disease (ded), neurotrophic ulcer, ocular burns, platelet-rich plasma (prp), platelet-rich fibrin (prf)

## Abstract

Introduction: The aim of this study was to evaluate the use of highly concentrated platelet-rich plasma (PRP) subconjunctival injections, in combination with eye drops (eye PRP, or E-PRP), in treating dry eye disease (DED) and the use of platelet-rich fibrin (PRF) membrane in treating corneal burns and neurotrophic ulcers for the restoration of the homeostasis of the tear film and the morphology and function of the cornea.

Methods: We studied 16 patients (n=32 eyes) with severe DED. The disease was graded as severe according to the Oxford Grading Scale. There were 12 males (75%) and four females (25%) with a mean age of 56 years; these patients were treated with monotherapy, which involved a single subconjunctival injection of 0.2-0.3 mL of PRP prepared from autologous blood, followed by application of autologous PRP eye drops four times a day for 15 days between September 2019 and December 2020 in the same geographic area. All patients gave written informed consent before undergoing the outlined treatment protocol. We evaluated best corrected visual acuity (BCVA), Schirmer test score, tear film breakup time (TBUT) and corneal staining with fluorescein (Oxford Grading Scale) before and after six to eight weeks of treatment. Subjective normalization was defined by a decrease in the Ocular Surface Disease Index (OSDI) score to 10 or less, an objective normalization of the TBUT to five to six seconds, improvement in the Schirmer test score and Oxford grading and the BCVA gain of at least one line in the vision chart (Snellen chart). Furthermore, we report on the results from different variants of platelet concentrate-based treatments in five cases of corneal diseases: neurotrophic ulcers and corneal burns due to different causes (e.g., chemical burns) using E-PRP and PRF membrane with regard to normalization of morphology and function.

Results: The OSDI score decreased to normalization in 75% of the patients (p=6.545 × 10^-15^). TBUT was restored significantly in 20 of 32 eyes from 2.78±0.55 to 5.43±0.71 (p=1.612 × 10^-24^). The Schirmer test score showed an improvement from 4.46±0.67 to 10.28±1.18 (p=3.411 × 10^-29^), and BCVA improved by 43.75%, from 0.15±0.19 to 0.09±0.16 (logMAR, p=0.2331). Oxford grading changed to Scale I in 75% and Scale 0 in 25% of the patients. No complications or adverse reactions occurred in the five cases of corneal injuries. We observed a restoration of the morphology and function of the cornea with PRP injections or PRF+PRP application in 7-12 days, depending on the severity of the initial finding.

Conclusion: PRP treatment is a new approach in ophthalmology with impressive results. Although patients show good compliance and acceptance of the treatment protocol, studies with larger sample sizes are needed to confirm and optimize its use.

## Introduction

Dry eye disease (DED) is a multifactorial disease of the ocular surface of the eye. It is characterized by a loss of homeostasis of the tear film and is accompanied by ocular symptoms in which tear film instability and hyperosmolarity, ocular surface inflammation damage and neurosensory abnormalities play an aetiological role [1,2]. The term “dry eye” encompasses various ocular disorders that can lead to dry eye conditions. Thus, global criteria are required for the diagnosis of DED, which does not necessarily identify a particular aetiology [1]. It has been established that most forms of dry eye exhibit similar features, such as burning, dryness, redness, itching or grittiness in the eyes [1-3].

Damage to the ocular surface is diagnosed using dyes such as fluorescein, rose Bengal and lissamine green. Tear film instability is established by measurements of tear film breakup time (TBUT). Tear film osmolality (hypertonicity) is known to be the common denominator between all forms of dry eye and is used as a marker as well. Our understanding of the mechanisms of the onset of DED and the evolution of related knowledge allows us to track the signs and ocular damage associated with the disease and the treatment and control of the evolution of different entities. The neurosensory network of the cornea plays a major role in the pathogenesis of DED [4]. Impairments of corneal sensory innervations cause reactions of both protective reflexes and trophic neuromodulators that are essential for the vitality, metabolism and regeneration of the ocular surface. The cornea is the most densely innervated tissue. The nerves stimulate wound healing and help maintain the regular anatomic integrity of the cornea and homeostasis [4,5]. DED is associated with pain, inflammation and nerve abnormalities. The ophthalmic division of the trigeminal nerve provides sensory innervation to the cornea. Neuropeptides’ role has been elucidated as they facilitate the crosstalk between immune and nervous systems and participate in suppressing inflammation in the cornea to preserve vision [4]. Any corneal injury or corneal nerve abnormalities may trigger a cellular and functional change within the trigeminal ganglion (TG). It has been demonstrated that DED results in decreased corneal density and loss of homeostasis [4-6].

DED is one of the most common ocular diseases. Between 10% and 40% of the patients who visit the ophthalmologist report symptoms of DED, which is a growing public health problem. The causes of DED are multifactorial, including age, hormonal issues, medications, contact lens use, smoking, laser refractive eye surgery, extended computer and smartphone use and working in low-relative-humidity air-conditioned places. A closed office environment can trigger DED, and allergies coexisting with autoimmune diseases can lead to symptoms related to DED [7-9]. The treatment prescribed for DED is the topical use of lubricants or preservative-free artificial tears associated in mild and severe forms with local active steroids. In severe and chronic cases, the use of cyclosporine is advocated; however, this treatment shows delayed results [4]. Blood-based treatments, such as autologous serum and platelet-rich plasma (PRP), show efficiency in mild and severe DED and can be used as first-line treatment in many situations, such as severe refractory DED, due to their contents of the growth factors, platelet-derived growth factor (PDGF), nerve growth factor (NGF), epidermal growth factor (EGF) and transforming growth factor beta (TGF-beta). The last three factors have neuroprotective effects, and PRP has similar osmolality, composition and pH to tear film [7-10].

PRP contains active cells, platelets that are involved in tissue repair, especially in DED, in nerve repairs due to NGF, more antioxidant properties, anti-inflammatory cytokines and other platelet derivatives, which are highly beneficial for the restoration and reconstruction of the ocular surface in DED [1-3,9,10].

PRF provides a fibrin scaffolding matrix with numerous growth factors, biochemical activators and cellular glues that have been shown to enhance the healing and regeneration of tissues [11-14]. PRF is a biomaterial and orthobiologic agent that promotes optimal healing with an activated fibrin matrix that helps glue damaged tissues, thereby giving the injured tissue a scaffolding on which to build new tissue. It also contains many cellular growth factors that drive tissue repair, such as PDGF, TGF-beta, VEGF, FGF NGF, EGF, insulin-like growth factor (IGF), bone morphogenetic proteins (BMP-2, BMP-7), thrombospondin and α2-macroglobulin. PRF matrix membrane can be used as a graft in conjunctivoplasty after pterygium surgery, with a low rate of complication and recurrences, and encouraging outcomes [15-19].

## Materials and methods

Study design

This interventional case series reports on 16 selected patients (n=32 eyes) with severe DED. (The disease was graded as severe according to the Oxford Grading Scale.) The patients were treated with monotherapy, which involved a single subconjunctival injection of 0.2-0.3 mL of highly concentrated eye PRP (E-PRP; PRP with fewer leukocytes), prepared from autologous blood, followed by application of autologous E-PRP eye drops four times a day for 15 days between September 2019 and December 2020 in the same geographic area. The study included 12 male patients (75%) and four female patients (25%), with a mean age of 56 years. We used the S-PRP WorldPRP cellular regeneration device and the TD5 centrifuge (S-PRP Biokit; Medi Sarang Co., Ltd, Seoul, South Korea).

Subjective normalization was defined by a decrease in the Ocular Surface Disease Index (OSDI) to a score of 10 or less, an objective normalization of the TBUT to five to six seconds, an improvement in the Schirmer test score and Oxford grading and the BCVA gain of one line in the vision chart.

An approval of the study was obtained from the Internal Ethical Committee of Ophthalmic Physicians (no. 23-01) of the International Multidisciplinary Society of Regenerative Medicine, Switzerland. All patients gave written informed consent before undergoing the outlined treatment protocol. Best corrected visual acuity (BCVA), Schirmer test score, TBUT and corneal staining with fluorescein (Oxford Grading Scale) were assessed before and after six to eight weeks of treatment.

Patient selection

Patient selection and enrollment took place at the Eye Center of Ophthalmology, Casablanca, Morocco, following the recommendations of the Helsinki Declaration. All patients underwent the following clinical examinations: visual acuity, slit lamp examination, Schirmer test under topical anaesthesia, fluorescein staining, fluorescein photography and TBUT assessment. Additionally, they filled out the OSDI questionnaire and recorded their scores, and the consent form with detailed information.

Inclusion and exclusion criteria

The selection of the patients was based on the severity of the objective finding (Oxford Grading Scale) and subjective symptoms such as pain and OSDI score. We included patients with severe symptoms and patients who had received other treatments without success. The selected corneal neurotrophic ulcer cases and ocular burn patients were previously treated elsewhere without any objective results. We excluded patients with allergic conditions, autoimmune diseases, patients with active arthritis, and those under treatment for pterygium, rosacea and sclera exposition. All patients were monitored on Day 0, Day 5, Day 30 and Day 60.

Pre- and post-intervention examinations

BCVA was assessed using refractions. The evaluation of Schirmer test scores, TBUT and corneal staining with fluorescein (Oxford Grading Scale) was performed before and after six to eight weeks of treatment. Subjective normalization was defined by a decrease in the OSDI score to 10 or less, objective normalization of the TBUT to five to six seconds, an improved Schirmer test score, an improvement in Oxford grading and a gain of one line in the BCVA as per the vision chart. All patients underwent a blood count examination to screen the platelet count level. Only patients with normal platelet levels were considered. Patients with ocular burns and neurotrophic ulcers were cleared for the treatment modalities and the expected results. Follow-up examinations were conducted for up to 30-60 days.

PRP processing and preparation

**Figure 1:**
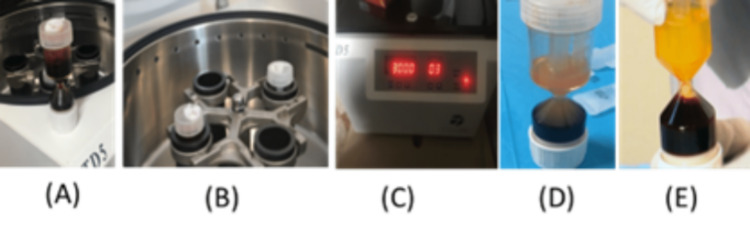
PRP yielding and separation of E-PRP and L-PRP (A) Blood sampling, (B) placing the device in the centrifugation machine, (C) setting the machine to 3000 rpm for three minutes (or in G force, RCF = (RPM)^2 ^× 1.118 × 10^-5 ^× r), (D) RBC, buffy coat (PRP), PPP, (E) separation of E-PRP and L-PRP RCF, relative centrifugal force; RPM, revolutions per minute; r, rotor radius; PRP, platelet-rich plasma; PPP, platelet-poor plasma; E-PRP, eye platelet-rich plasma; L-PRP, leukocyte- and platelet-rich plasma

The following protocol was followed for PRP preparation: whole blood was collected by venipuncture prior to injection; 20 cc of blood was anticoagulated at a ratio of 1:10 (anticoagulant:blood) and centrifuged at 3000 rpm for three minutes in a special closed-circuit device (PRP WorldPRP cellular regeneration device and the TD5 centrifuge). This method employs a clock house device that separates platelet-poor plasma (PPP), PRP, leukocytes and red blood cells and allows precise sampling of highly concentrated PRP with a closed system (Figure 1). The buffy coat can be used separately to divide leucocyte- and platelet-rich plasma (L-PRP) from highly concentrated PRP. The concentration is at least 10 times higher than the baseline count found in native blood.

**Figure 2:**
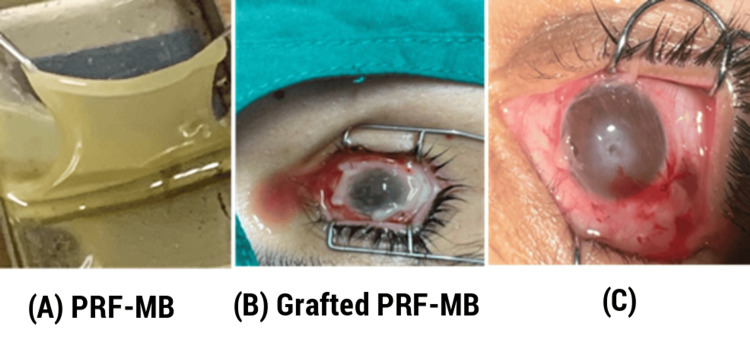
(A) PRF membrane (PRF-MB) prepared from patient's blood, (B, C) membrane graft sutured in the conjunctiva covering the whole corneal surface PRF, platelet-rich fibrin

PRF is a compact variant of the platelet concentrate trapped in a fibrin matrix obtained by special methods of centrifugation of non-citrated blood in a tube or kit frozen at -20°C and put in an extended sterile cupule. It yields a membrane that can be used for surface treatments (Figure 2). The PRF membrane acts as a scaffold for cellular grafts, mirroring properties similar to an amniotic membrane with sustained releases of bioactive and growth factors that contribute to driving the regeneration process.

Procedure

All patients were administered subconjunctival injections of 0.2-0.3 mL of E-PRP under topical anaesthesia, using oxybuprocaine chlorhydrate (Cebesine 0.4%) eye drops after disinfecting the eye with 10% povidone-iodine (Betadine 10%) for three minutes and rinsing with the saline solution. The residual PRP+PPP collected from the device was filled in two sterile eye droppers to be used four times per day. After injecting, for 14 days, the first dropper was conserved at +4°C for one week, and the second dropper was frozen at -20°C until consumption of the first eye dropper and used similarly.

Outcomes

A total of 68 weeks later, we assessed the following parameters after fluorescein staining and fluorescein staining photography: OSDI, BCVA, TBUT, Schirmer test score and Oxford grading. The OSDI score decreased to normalization in 75% of the patients (p=6.545 × 10^-15^). TBUT was restored significantly in 20 of 32 eyes from 2.78±0.55 to 5.43±0.71 (p=1.612 × 10^-24^). The Schirmer test score showed an improvement from 4.46±0.67 to 10.28±1.18 (p=3.411 × 10^-29^). BCVA (gain of one line or more in visual acuity) improved by 43.75% from 0.15±0.19 to 0.09±0.16 (logMAR, p=0.2331). The Oxford grading changed to Scale I in 75% and to Scale 0 in 25% of the patients.

Statistical analysis

The data obtained were analysed using IBM SPSS Statistics, version 20.0 (IBM Corp., Armonk, NY). Measurable values were expressed as means ± standard deviations and compared using an independent sample t-test. Non-measurable data were expressed as percentages.

## Results

Demographic data of the studied patients

We included 16 patients, 12 males and 4 females, with a mean age of 56 years (Table 1).

**Table 1:** Demographic data of the studied patients (n=16)

	Variable	Studied patients (n=16)
Age (years)	Mean±SD	53.75±14.34
	Range	28-73
	Median	56
Sex	Male	12 (75%)
	Female	4 (25%)

OSDI

**Figure 3:**
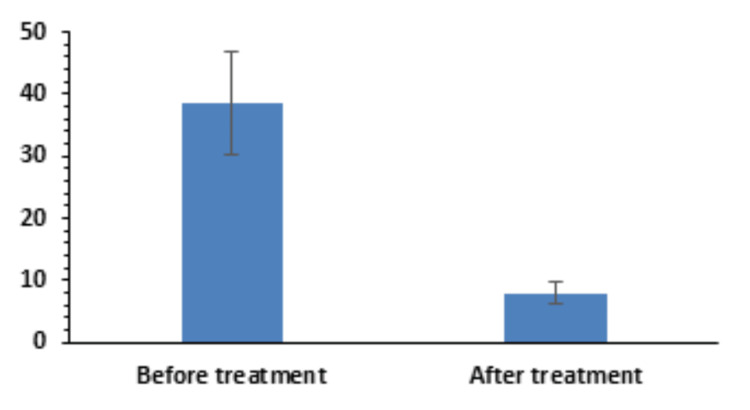
Pre- and post-treatment evaluation of OSDI scores OSDI, Ocular Surface Disease Index

There was an improvement in the OSDI score from 38.62±8.42 to 8.01±1.62 (Figure 3), which was statistically significant (p=6.545 × 10^-15^).

TBUT

**Figure 4:**
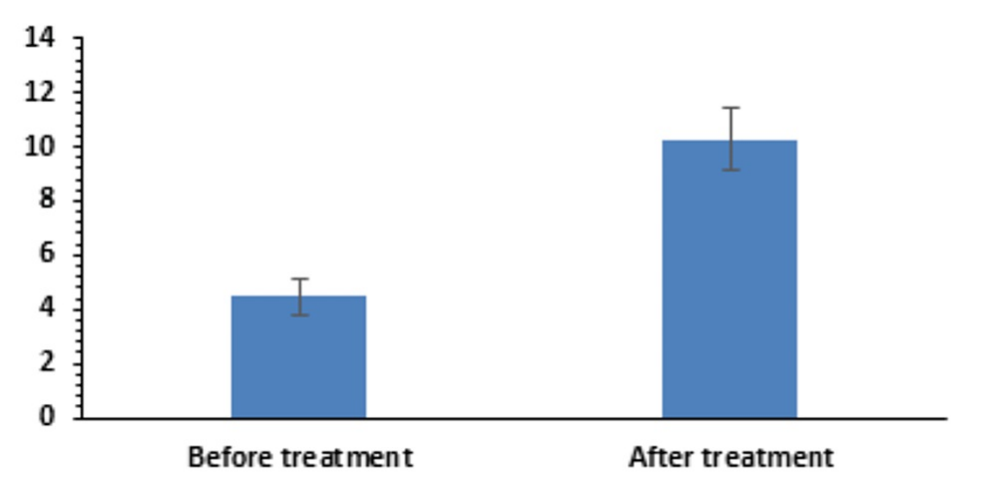
Pre- and post-treatment comparison of TBUT in patients who received PRP TBUT, tear film breakup time; PRP, platelet-rich plasma

There was a prompt improvement in TBUT from 2.78±0.55 before treatment to 5.43±0.71 seconds after treatment (p=1.612 × 10^-24^), which was statistically significant (Figure 4).

Oxford Grading Scale

**Figure 5:**
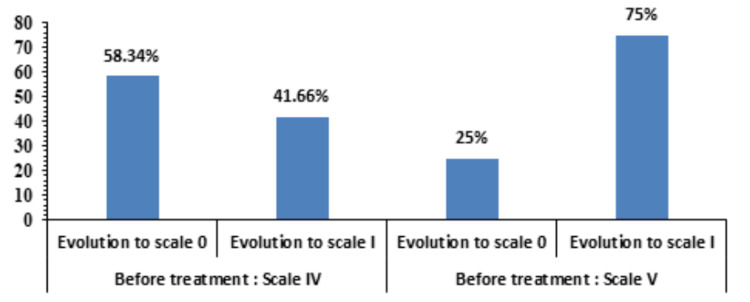
Pre- and post-treatment comparison of Oxford grading

After treatment, the Oxford Grading Scale improved (Scale 0) in the majority of the cases with moderate DED (Scale IV). However, in severe cases (Scale V), only 25% of patients improved to Scale 0, while 75% reversed to Scale I (Figure 5).

Schirmer test score

**Figure 6:**
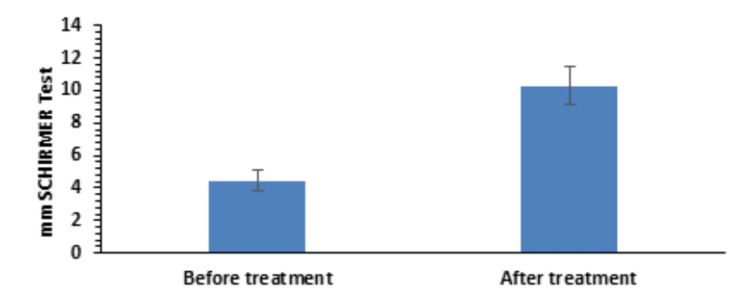
Pre- and post-treatment Schirmer test results

Regarding the Schirmer test score, there was an improvement from 4.46±0.67 to 10.28±1.18 (Figure 6), which was statistically significant (p=3.411 × 10^-29^).

Case series about the efficacy of PRP and PRF treatment in neurotrophic ulcers and corneal burns

**Figure 7:**
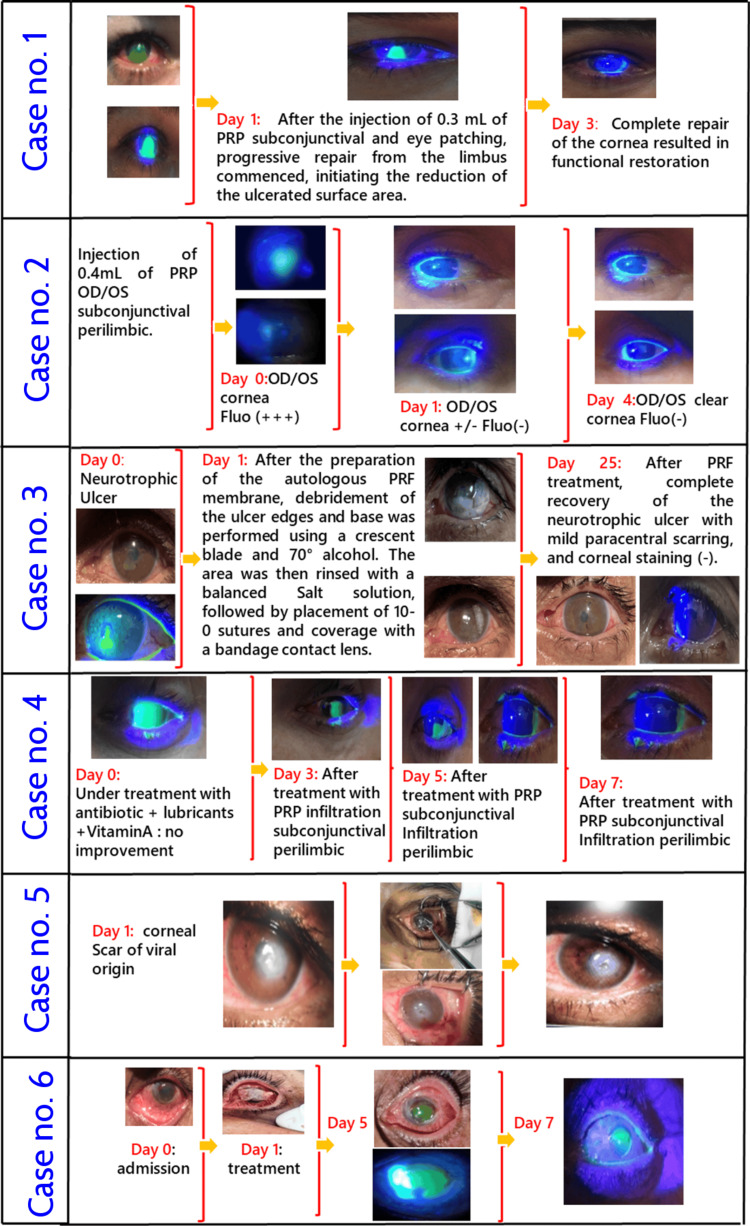
Casuistic PRP and PRF treatments in selected cases of neurotrophic ulcers and corneal burns PRP, platelet-rich plasma; PRF, platelet-rich fibrin; Fluo, fluorescein staining

Table 2 and Figure 7 summarize all the cases studied with the evolution of treatment of neurotrophic ulcers and corneal burns. Cases of ocular burns of different etiologies received a subconjunctival PRP injection. The recovery started on Day 3; depending on the severity of the lesions, the results were consistent with a complete healing, except for a patient with a caustic burn with severe and extensive lesions who required a PRF membrane with a longer recovery time, and the use of topical adjuvant treatments. Case number 3 was a patient with a neurotrophic ulcer and case number 5 was with a viral keratitis scar. Both cases showed good results in four weeks, with recovery and a rapid decrease in inflammation.

**Table 2:** Casuistic PRP and PRF treatments in selected cases of neurotrophic ulcers and corneal burns PRP, platelet-rich plasma; PRF, platelet-rich fibrin

Case	Description	Course of PRP and PRF treatment in neurotrophic ulcers and corneal burns
No. 1	A 34-year-old man with chemical ocular burns (projection of cement powder in his right eye), treated with antibiotics and autologous serum for one week without results, came to us with this condition (PRP treatment).	Complete functional and morphological recovery with corneal staining (-)
No. 2	A 52-year-old woman with depression-related articular pain; dry eyes were treated with all medical protocols without success. She complained about pain and blurred vision. After PRP injection, normalization of the corneal surface occurred with no pain and no blurred vision (PRP treatment).	Complete recovery with better functional improvements with corneal staining (-)
No. 3	A 67-year-old diabetic patient with a chronic neurotrophic ulcer, previously treated without success, underwent PRF membrane treatment after debridement of the base and edges of the ulcer using a crescent blade and alcohol, followed by rinsing with a balanced salt solution. Complete recovery was observed.	Prompt reduction of the ulcer, along with decreased depth and flattening of the edges. Recovery was reached after 24 days
No. 4	A 55-year-old man, a security officer, accidentally exposed to paint thinner in his left eye; treated for one week without any positive results (PRP treatment).	Complete recovery with 100% functional conditions with corneal staining (-)
No. 5	A 34-year-old woman treated for viral keratitis. To prepare for a keratoplasty (corneal graft), we initially applied a PRF membrane covering to reduce inflammation, ensuring the success of the corneal graft.	Decrease in the scarred tissue and inactivity; the rest of the cornea cleared; corneal graft could be done
No. 6	A 45-year-old woman with a caustic ocular burn (quicklime) treated for 8 weeks without results; treated with a PRF membrane graft (solid PRF) on Day 0, Day 5 and Day 7.	Decrease in the extensive lesions and stabilisation, with corneal healing reaching at 95% approximately

These results justify the early implementation of blood-based treatments in different situations, such as persistent corneal erosions, corneal burns and neurotrophic ulcers; however, large-sample studies are needed to assess the efficacy.

## Discussion

In this study, we showed that the treatment of severe DED with optimized PRP led to prompt improvement of the outcomes in the majority of the cases; this improvement was presented in terms of the OSDI score, TBUT and Oxford grading (Table 3, Appendices). A previous study reported a significant decrease (75%) in irritation symptoms (OSDI); another clinical trial also showed a 40% decrease in the discomfort score and significant (55%) improvements in visual acuity (p<0.05) [11,12]. Another study confirmed significant improvements of 87.5% after six weeks of PRP eye drop treatment; eye symptoms improved with a decrease in corneal fluorescein staining in 76.1% of patients, and 28.8% of patients showed improvement in at least one line of BCVA. Furthermore, the OSDI score decreased significantly after treatment [12].

Administering a subconjunctival injection of PRP adds value, complemented by the subsequent use of PRP eye drops, especially in some seemingly helpless situations where the implementation of a therapeutic adjuvant is justified and needed. This finding correlates with a previous study on animal models that used the same protocol (subconjunctival injection PRP in several difficult and compromised clinical issues) [13].

The cases of ocular burns treated with injections of PRP and the PRF membrane with sustained release of growth factors and matrix proteins showed impressive results in two different situations (Case no. 1 and Case no. 6). Further studies showed good outcomes with PRP eye drops as a complement to the classical treatment and artificial tears [14]. Other treatment regimens using the pharmacologic regenerating agent (RGTA) showed success in neurotrophic ulcers for selected cases (33%) and failures in 67% of cases in six weeks [10].

Case number 4, for example, was treated for one week without any improvement (Figure 7). However, treatment with a single injection combined with PRP eye drops led to rapid healing in seven days with a total morphologic reconstruction of the surface of the cornea and its function. Case number 6 indicates that research must be conducted to optimize the application of the combination of PRP and PRF membrane. We could observe the remarkable healing process in only one week. The cornea became clear with a visible Purkinje reflex after eight weeks of treatment with the classical protocol, without a positive response. In this situation, every optimization effort must be made to manage this emergency.

## Conclusions

Growth factors and platelets play an essential role in the different stages of cell and tissue repair. Autologous platelet concentrates and growth factors or PRP can potentially show effective therapeutic benefits, depending on the pathophysiological mechanisms targeted and the severity of the lesions.

In selected cases of corneal diseases with different causes, the use of highly concentrated PRP and PRF membrane results in good restoration of homeostasis of the tear film and the restoration of morphological and functional outcomes. PRP treatment is a new approach in ophthalmology. It is a simple treatment with impressive results and repeatable without any adverse reactions. Also, there is good compliance and acceptance of the treatment protocol by the patients. However, studies with larger sample sizes are needed to confirm and optimize its use.

## Appendices

Data recorded

**Table 3:** Data recorded before and 60 days after treatment OSDI, Ocular Surface Disease Index; TBUT: tear film breakup time; BCVA: best corrected visual acuity; OXFG: Oxford grading

	OSDI		BCVA (Snellen chart)		TBUT		OXFG		Schirmer test score	
Patient	Before	After	Before	After	Before	After	Before	After	Before	After
No. 1	38.5	8	0.9/0.9	1/1	2/2	5/5	IV	0	4/4	12/12
No. 2	48	10	0.8/0.9	1/1	2/2	6/6	V	0	5/5	12/12
No. 3	37.5	8	0.3/0.9	0.4/1	2/3	6/6	IV	I	4/4	12/12
No. 4	48	8	0.5/0.5	0.6/0.5	2/3	6/6	IV	I	6/6	12/12
No. 5	27	9	0.4/0.5	0.5/0.5	3/2	6/6	IV	I	4/4	12/12
No. 6	47	8	0.9/0.9	1,00	3/3	6/6	V	0	4/4	12/12
No. 7	30	8	0.2/0.6	0.3/0.6	3/3	4/4	V	II	5/5	12/12
No. 8	27	8	1/1	1/1	3/3	6/6	IV	0	5/5	12/12
No. 9	30	7	1/1	1/1	3/3	6/6	IV	0	4/4	12/12
No. 10	27	10	0.8/0.4	0.9/0.4	4/4	6/4	V	I	3.5/3.5	12/12
No. 11	48	7	0.9/0.9	1/1	3/3	5/5	V	I	4/4	12/12
No. 12	40	7	0.9/1	1/1	2/2	6/6	V	I	5/5	12/12
No. 13	48	9	0.9/1	0.9/1	3/3	5/5	IV	I	4/4	12/12
No. 14	35	10	0.9/0.9	1/1	3/3	5/5	IV	0	5/5	12/12
No. 15	47	10	0.9/0.9	1/1	3/3	6/6	IV	0	4/4	12/12
No. 16	40	10	0.9/1	1/1	3/3	6/6	IV	0	4/4	12/12
